# Revising the ABIDE MCI to dementia prediction model for automated cerebrospinal fluid assays

**DOI:** 10.1002/alz.71192

**Published:** 2026-02-09

**Authors:** Pieter J. van der Veere, Argonde C. van Harten, Ingrid S. van Maurik, Charlotte E. Teunissen, Frederik Barkhof, Stephanie J. B. Vos, Lutz Froelich, Johannes Kornhuber, Jens Wiltfang, Wolfgang Maier, Oliver Peters, Eckart Rüther, Giovanni B. Frisoni, Luiza Spiru, Yvonne Freund‐Levi, Åsa K. Wallin, Harald Hampel, Magda Tsolaki, Iwona Kłoszewska, Patrizia Mecocci, Bruno Vellas, Simon Lovestone, Samantha Galluzzi, Sanna‐Kaisa Herukka, Isabel Santana, I. Baldeiras, Alexandre de Mendonca, Dina Silva, Gael Chetelat, Géraldine Poisnel, Pieter Jelle Visser, Sterling C. Johnson, Erik Stormrud, Oskar Hansson, Sebastian Palmqvist, Gerard Piñol‐Ripoll, for the Alzheimer's Disease Neuroimaging Initiative, Johannes Berkhof, Wiesje M. van der Flier

**Affiliations:** ^1^ Amsterdam Neuroscience Amsterdam The Netherlands; ^2^ Alzheimer Center Amsterdam Department of Neurology Vrije Universiteit Amsterdam Amsterdam UMC location VUmc Amsterdam the Netherlands; ^3^ Department of Epidemiology and Data Science Amsterdam UMC Amsterdam the Netherlands; ^4^ Neurochemistry Laboratory Department of Laboratory Medicine Amsterdam Neuroscience Vrije Universiteit Amsterdam Amsterdam UMC Amsterdam the Netherlands; ^5^ Northwest Academy Northwest Clinics Alkmaar Alkmaar The Netherlands; ^6^ UCL Hawkes Institute Dept of Medical Physics University College London London UK; ^7^ Department of Radiology & Nuclear Medicine Vrije Universiteit Amsterdam Amsterdam University Medical Center Amsterdam the Netherlands; ^8^ Department of Psychiatry and Neuropsychology Maastricht University School for Mental Health and Neuroscience Alzheimer Centre Limburg Maastricht Netherlands; ^9^ Department of Geriatric Psychiatry Zentralinstitut für Seelische Gesundheit Medical Faculty Mannheim University of Heidelberg Mannheim Germany; ^10^ Department of Psychiatry and Psychotherapy Friedrich‐Alexander University of Erlangen‐Nürnberg Erlangen Germany; ^11^ Department of Psychiatry and Psychotherapy University Medical Center Georg‐August‐University Göttingen Germany; ^12^ German Center for Neurodegenerative Diseases Göttingen Germany; ^13^ iBiMED Medical Sciences Department University of Aveiro Campus Universitário de Santiago Aveiro Portugal; ^14^ Department of Neurodegenerative Diseases and Gerontopsychiatry University of Bonn German Center for Neurodegenerative Diseases Bonn Germany; ^15^ Department of Psychiatry Charité—Universitätsmedizin Berlin corporate member of Freie Universität Berlin Humboldt‐Universität zu Berlin, and Berlin Institute of Health Berlin Germany; ^16^ German Center for Neurodegenerative Diseases Berlin Germany; ^17^ Memory Clinic University Hospital and University of Geneva Genève Switzerland; ^18^ Geriatrics Gerontology Old Age Psychiatry and Longevity Medicine ‐ Clinical Department Carol Davila University of Medicine and Pharmacy Saint Luke's Clinical Hospital Bucharest Romania; ^19^ Memory Clinic Brain/Mental Health and Longevity Medicine Ana Aslan International Foundation Bucharest Romania; ^20^ School of Medical Sciences Örebro University Örebro Sweden; ^21^ Division of Clinical Geriatrics Center for Alzheimer Research Department of Neurobiology Care Sciences and Society Karolinska Institutet Stokholm Sweden; ^22^ Department of Old Age Psychiatry Psychology and Neuroscience King's College London London UK; ^23^ Department of Clinical Sciences Malmo Cognitive Disorder Research Unit Lund University, Malmo Lund Sweden; ^24^ Alzheimer Precision Medicine (APM), AP‐HP Sorbonne University Pitié‐Salpêtrière Hospital Paris France; ^25^ 1st Department of Neurology Aristotle University of Thessaloniki Alzheimer Hellas Lab of Neurodegenerative Diseases Center for Interdisciplinary Research and Innovation Aristotle University of Thessaloniki, Makedonia Thessaloniki Greece; ^26^ Department of Geriatric Psychiatry and Psychotic Disorders Medical University of Lodz Lodz Poland; ^27^ Institute of Gerontology and Geriatrics Department of Medicine and Surgery University of Perugia Santa Maria della Misericordia Hospital Perugia Italy; ^28^ I.H.U. HealthAge Toulouse University Hospital Toulouse France; ^29^ Department of Psychiatry University of Oxford Oxford UK; ^30^ IRCCS Istituto Centro San Giovanni di Dio Fatebenefratelli, BS Brescia Italy; ^31^ Institute of Clinical Medicine Neurology University of Eastern Finland and Neurocenter Neurology Kuopio University Hospital Kuopi Finland; ^32^ Center for Neuroscience and Cell Biology Faculty of Medicine University of Coimbra Rua Larga ‐ Faculdade de Medicina Coimbra Portugal; ^33^ Department of Neurology Hospitais da Universidade de Coimbra ULS Coimbra, Praceta Professor Mota Pinto Coimbra Portugal; ^34^ Faculty of Medicine University of Coimbra Coimbra Portugal; ^35^ Faculty of Medicine University of Lisboa Lisboa Portugal; ^36^ Institute of Molecular Medicine University of Lisboa Lisboa Portugal; ^37^ Centre for Biomedical Research Universidade do Algarve Faro Portugal; ^38^ Normandie Univ UNICAEN INSERM Caen France; ^39^ Department of Medicine University of Wisconsin‐Madison Madison Wisconsin USA; ^40^ Wisconsin's Alzheimer's Disease Research Center Madison Wisconsin USA; ^41^ Memory Clinic Skåne University Hospital Malmö Sweden; ^42^ Unitat Trastorns Cognitius Clinical Neuroscience Research Hospital Universitari Santa Maria Lleida Spain; ^43^ Alzheimer's Disease and Other Cognitive Disorders Unit Neurology Service Hospital Clínic de Barcelona Fundació de Recerca Clínic ‐ Institut d'Investigacions Biomèdiques August Pi i Sunyer (IDIBAPS) Universitat de Barcelona Barcelona Spain; ^44^ Amsterdam Public Health Amsterdam the Netherlands

**Keywords:** Alzheimer's disease, automated cerebrospinal fluid assays, cerebrospinal fluid, dementia, mild cognitive dementia, prediction

## Abstract

**INTRODUCTION:**

Automated cerebrospinal fluid (CSF) biomarker assays have largely replaced manual immunoassays for measuring amyloid pathology in CSF. We refitted and validated the ABIDE model, predicting progression from mild cognitive impairment (MCI) to dementia, with CSF measurements from the automated Elecsys platform.

**METHODS:**

We included 2413 MCI participants (998 [41%] amyloid‐positive) from seven observational cohorts. Elecsys was used in 958 (40%) participants. The parameters of the previous ABIDE Cox model were re‐estimated. Model discrimination and calibration were evaluated with leave‐one‐cohort‐out cross‐validation.

**RESULTS:**

During follow‐up, 1034 (42%; 585 [58%] amyloid‐positive) participants developed dementia. Discrimination was good with Harrell's C of 0.70 (95% confidence interval [CI]: 0.66–0.73). Calibration was good in the total population and amyloid‐positive subgroup, with substantial predicted progression risks for all amyloid‐positive participants.

**DISCUSSION:**

We refitted the ABIDE model, predicting MCI to dementia progression, with automated CSF measurements. The model was well calibrated in amyloid‐positive patients and may support clinical discussions regarding ATTs.

## Highlights

- We updated the ABIDE mild cognitive impairment (MCI) to dementia prediction model.
- The model has good discrimination with a Harrell's C of 0.70 (95% confidence interval [CI]: 0.66–0.73).
- The model is well calibrated in the total population and amyloid‐positive subgroup.

## 1 INTRODUCTION

Dementia is a leading cause of lost healthy life‐years,^1^ and the prevalence is projected to rise in the upcoming decades due to population ageing. As neurodegeneration gradually develops, patients often have mild cognitive problems (i.e. mild cognitive impairment [MCI]) for a period before the onset of dementia. Patients increasingly present with MCI at memory clinics, worrying that their problems may worsen to dementia in the future.^2^ The ABIDE model is a widely cited model predicting a patient's probability to progress from MCI to dementia over time.^3^ However, since its publication in 2019, two important developments warrant an updated model. First, the cerebrospinal fluid (CSF) Aβ1‐42 and pTau181 measurements used in the ABIDE model were largely performed using manual INNOTEST assays. Since then, automated CSF measurement assays like Elecsys have become standard. This has strongly reduced within and cross‐site measurement variability, improved the generalizability of CSF measurements, and introduced use of the pTau181/Aβ1‐42‐ratio,^4^ indicating a potentially altered relationship between CSF biomarkers for Innotest and Elecsys. Second, the first amyloid‐targeting therapies (ATTs) for Alzheimer's disease (AD) have come to market. This has contributed to the adoption of a biological definition of AD,^5^ as ATTs require confirmation of amyloid pathology for treatment eligibility.^6^ It has also reinforced the need for personalized risk predictions to support doctor and patients discussions regarding initiation of treatment.

We aimed to update the ABIDE prediction model for two objectives: (1) refit the model based on a larger proportion of Elecsys CSF assay measurements, and (2) evaluate the performance of the ABIDE model in amyloid‐positive patients, who are potentially eligible for ATTs.

## 2 METHODS

### 2.1 Study design and participants

Aligning with the original design,^3^ we included participants from single and multicenter cohorts in Europe and the United States diagnosed with MCI at baseline, with a baseline Mini‐Mental State Examination (MMSE), either a baseline magnetic resonance imaging (MRI) hippocampal volume measurement or CSF measurements of Aβ1‐42 and pTau181, and at least six months of follow‐up. This resulted in the following number of participants per cohort: Amsterdam Dementia Cohort (ADC; *n* = 689),^7^ Alzheimer's Disease Neuroimaging Initiative (ADNI; *n* = 544),^8^ Biomarkers For Identifying Neurodegenerative Disorders Early and Reliably Study 1 (BioFINDER‐1; *n* = 212; NCT01208675),^9^ European Medical Information Framework for Alzheimer's Disease (EMIF‐AD; *n* = 809; composed of DESCRIPA, AddNeuroMed, German Dementia Competence Network, IMAP, Brescia, Coimbra, Kuopio, and Lisbon),^10^ Lleida cohort (*n* = 88),^11^ National Alzheimer's Coordinating Centre (NACC) Uniform Data Set (*n* = 63),^12^ and the Wisconsin Alzheimer's Disease Research Centre (W‐ADRC; *n* = 8).^13^ See Supplemental Table 1 for cohort characteristics, and Supplemental Table 2 for baseline characteristics of the newly included participants (*n* = 477) and those with additional follow‐up (*n* = 194). Of these cohorts, W‐ADRC, NACC, and ADNI are research cohorts, and the others are memory clinic cohorts. Due to their respective sizes, NACC and W‐ADRC were combined into a USA‐other cohort. From ADNI, only “late‐MCI” participants were selected as “early‐MCI” has a dementia progression rate similar to cognitively normal individuals,^14^ which is very different from all other included cohorts. An amyloid‐positive subgroup was defined based on CSF (see Supplemental Methods for cut‐offs).

All participants gave written informed consent for participation in the original studies. This study is reported in accordance with the Transparent Reporting of a multivariable prediction model for Individual Prognosis Or Diagnosis (TRIPOD) guideline.

### 2.2 Predictors

All predictors were collected at baseline. A detailed overview of the measurement method per study was provided in the previous ABIDE study and the supplement.^3^ In brief, CSF phosphorylated tau181 (pTau181; available in 1540 [64%] participants) and amyloid β1‐42 (Aβ1‐42; available in 1518 [63%] participants) were measured with the Elecsys (*n* = 958; 61%), Innotest (*n* = 577; 37%), and Luminex (*n* = 41; 3%) assays. Values were bridged between platforms to represent Elecsys values. Further predictors were hippocampal volume (available in 1985 [82%] participants), age, sex, and MMSE. Missing CSF and MRI values were imputed using multiple imputation with chained equations with details in the Supplemental Methods. For the analyses, Aβ1‐42 and pTau181 were log‐transformed and all continuous variables were standardized.

**RESEARCH IN CONTEXT**

1. **Systematic review**: We searched PubMed for articles on the prediction of mild cognitive impairment (MCI) to dementia conversion using cerebrospinal fluid (CSF) biomarkers measured with automated assays. While several studies investigated this, the number of included MCI patients and identified conversions during follow‐up were relatively small.
2. **Interpretation**: To our best knowledge, this study includes the largest number of MCI patients with automated CSF measurements, as well as the most MCI to dementia conversions. We updated the ABIDE MCI to dementia prediction model using additional automated CSF measurements performed using the Elecsys assay. The prediction model is well calibrated across the utilized assays and in the amyloid‐positive subpopulation.
3. **Future directions**: Our findings bring the ABIDE prediction model in line with current clinical practice, where automated CSF measurements have become standard. Novel predictors explaining additional variation in progression patterns are needed to further improve the predictive accuracy.

### 2.3 Outcome

The primary outcome was clinical progression to any type of dementia during follow‐up.

### 2.4 Statistical analysis

The coefficients of the previously published ABIDE Cox model were re‐estimated on MCI participants pooled from the cohorts,^3^ hereafter called the refitted ABIDE model. Model performance was evaluated using leave‐one‐cohort‐out cross‐validation, where iteratively one cohort served as a test set in which predictions were made with models developed in the remaining cohorts. Discrimination in the test cohorts was evaluated using Harrell's C. Calibration of the models was assessed in the test cohorts by comparing the predicted and observed MCI to dementia progression risk in four risk groups defined by predicted risk percentile thresholds: low (< 16^th^ percentile), low‐medium (P16–P50), medium‐high (P50–P84), and high (> P84). The predicted median progression time in each risk group was calculated with bootstrapped confidence intervals. Additionally, mean absolute errors were calculated using pseudo‐observations with bootstrapped confidence intervals,^15^ which can be interpreted as the mean difference between the time at which an individual has a predicted progression risk of 50% and the observed event time. Calibration and discrimination of the refitted ABIDE model were also assessed in the amyloid‐positive subgroup, in each cohort and for each CSF assay.

Analyses were performed with R 4.3.2. Prediction models will be incorporated in www.adappt.health, a platform with information on AD for clinicians.

## 3 RESULTS

Of the 2413 MCI participants (mean ± SD age: 69 ± 8 years, 1373 [57%] female), 1034 (43%) developed dementia (Table 1). Of the 998 (41%) amyloid‐positive participants, 585 (59%) developed dementia, and they had similar age, sex, MMSE, and MRI hippocampus volume distributions as those in the total population (Supplemental Table 3).

**TABLE 1:** Baseline characteristics.

	Cohorts						
Parameter	Total (*n* = 2413)	EMIF‐AD (*n* = 809)	ADC (*n* = 689)	ADNI (*n* = 544)	BioFINDER‐1 (*n* = 212)	Lleida (*n* = 88)	USA other (*n* = 71)^†^
Follow‐up time (years)—median (IQR)	2.1 (1.3–3.3)	2.0 (1.0–3.0)	2.4 (1.4–4.0)	2.2 (1.5–4.0)	2.4 (2.1–4.1)	2.0 (0.8–4.5)	2.2 (1.2–3.4)
Participants progressed to dementia, *n* (%)	1034 (42.9)	247 (30.5)	296 (43.0)	272 (50.0)	127 (59.9)	46 (52.3)	46 (64.8)
Age at baseline (years)—mean ± SD	69 ± 8	69 ± 8	66 ± 7	74 ± 8	71 ± 5	74 ± 6	76 ± 9
Female—*n* (%)	1373 (56.9)	389 (48.1)	443 (64.3)	335 (61.6)	126 (59.4)	39 (44.3)	41 (57.7)
MMSE at baseline—mean ± SD	26.8 ± 2.3	26.9 ± 2.4	26.6 ± 2.3	27.2 ± 1.9	27.2 ± 1.9	25.2 ± 3.0	26.9 ± 2.2
Amyloid β1‐42 (pg/ml)—median (IQR)	822 (581–1256)	850 (529–1348)	880 (639–1382)	694 (555–935)	877 (645–1346)	753 (547–1144)	761 (516–1402)
Phosphorylated tau181 (pg/ml)—median (IQR)	23.8 (16.2–34.3)	20.5 (13.8–32.7)	24.4 (16.7–34.2)	27.8 (19.3–37.8)	21.4 (15.9–32.0)	21.2 (16.1–31.1)	24.9 (15.0–38.2)
Amyloid‐positive^*^—*n* (%)	998 (41.4)	224 (27.7)	344 (49.9)	225 (41.4)	109 (51.4)	55 (62.5)	41 (57.7)
MRI hippocampal volume (ml)—mean ± SD	6.8 ± 1.2	6.8 ± 1.2	7.2 ± 1.1	6.6 ± 1.1	6.2 ± 1.0	NA	5.8 ± 1.1
**CSF platform—*n* (%)**							
Elecsys	958 (60.8)	NA	357 (60.7)	310 (100)	195 (100)	88 (100)	8 (16.3)
Innotest	577 (36.6)	346 (100)	231 (39.3)	NA	NA	NA	NA
Luminex	41 (2.6)	NA	NA	NA	NA	NA	41 (83.7)

Abbreviation: ADC, Amsterdam Dementia Cohort; ADNI, Alzheimer's Disease Neuroimaging Initiative; BioFINDER, Biomarkers For Identifying Neurodegenerative Disorders Early and Reliably Study; EMIF‐AD, European Medical Information Framework for Alzheimer's Disease; MMSE, Mini‐Mental State Examination; MRI, magnetic resonance imaging; NACC, National Alzheimer's Coordinating Centre; SD, standard deviation; W‐ADRC, Wisconsin Alzheimer's Disease Research Centre.

* Elecsys pTau181/Aβ1‐42 ratio > 0.020, Luminex Aβ1‐42 < 250pg/ml, Innotest Aβ1‐42 < 813pg/ml. †Contains the NACC and W‐ADRC cohorts. NA was used if no data was present.

**FIGURE 1:**
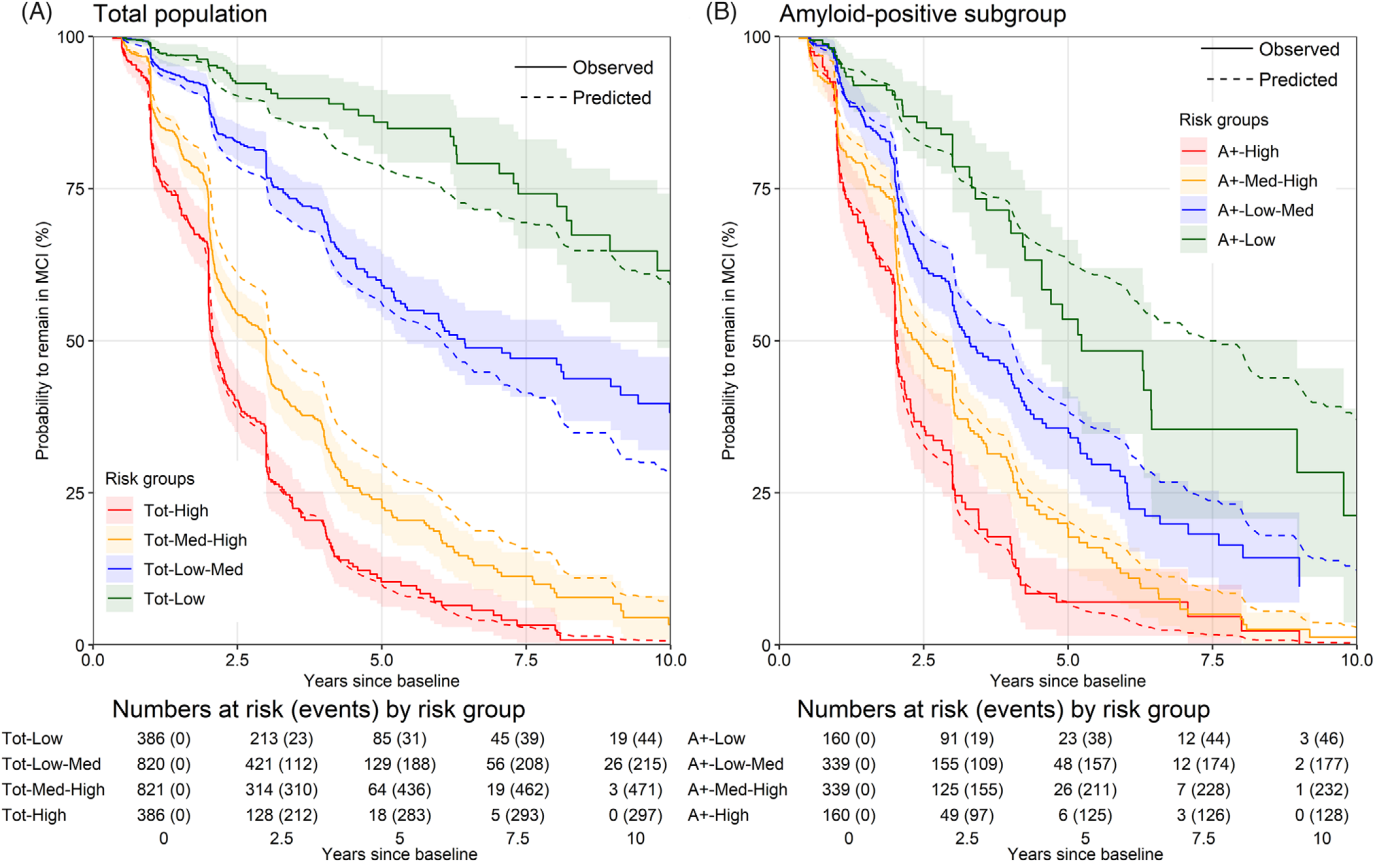
Risk groups in the total population and amyloid‐positive subgroup. The predicted survival curves are the average predicted survival probabilities of individuals in each risk group. Leave‐on‐cohort‐out cross‐validation was used to make the predictions where iteratively one cohort was selected as “test” set in which predictions were made using a model develop in the other cohorts. The risk groups are based on the distribution of predicted risks: low (< P16), low‐medium (P16–P50), medium‐high (P50–P84), high (> P84). The shaded areas correspond to the 95% confidence intervals of the observed survival probabilities.

The coefficients of the refitted ABIDE model, predicting progression from MCI to dementia in all MCI patients, did not substantially change compared to the old model (Table 2; Supplemental Table 4), except for a larger interaction effect CSF Aβ1‐42 and pTau181 in the refitted model. Cross‐validation showed a Harrell's C of 0.70 (95% confidence interval [CI]: 0.66–0.73; Supplemental Figure 1), which is in line with the previous model's Harrell's C of 0.74 (95% CI: 0.71–0.76).^3^ The cross‐validated predicted and observed MCI to dementia progression probabilities largely overlapped, except for a slight overestimation of risk in the low‐risk group, indicating acceptable calibration (Figure 1A). In the amyloid‐positive subgroup, the cross‐validation showed a Harrell's C of 0.63 (95% CI: 0.58–0.69; Supplemental Figure 2). The lower discrimination in the amyloid‐positive subgroup is in line with the reduced heterogeneity, that is a uniformly worse prognosis compared to the total population. The predicted and observed progression probabilities overlapped in all amyloid‐positive risk groups, indicating good calibration (Figure 1B). Additionally, the prediction error in the amyloid‐positive subgroup was 2.54 (95% CI: 2.36–2.72) compared to 3.92 (95% CI: 3.77–4.06) years in the total population, indicating that the lower discrimination of the model in this subgroup did not result in a larger predictive error. Calibration and discrimination by cohort and CSF assay are reported in the supplemental results (Supplemental Figures 3 and 4).

**TABLE 2:** Partial regression coefficients of the refitted ABIDE model.

Parameter	Partial regression coefficients (95% CI)
Amyloid β1‐42, per SD change in log pg/ml	−0.33 (−0.40–−0.25)
Phosphorylated tau181, per SD change in log pg/ml	0.33 (0.25–0.41)
MRI hippocampal volume, per SD change in ml	−0.37 (−0.45–−0.29)
Age, per SD	−0.05 (−0.12–0.03)
MMSE, per SD	−0.23 (−0.30–−0.17)
Interactions	
Amyloid β * phosphorylated tau181	0.07 (−0.03–0.17)
Amyloid β * age	0.04 (−0.03–0.12)
Phosphorylated tau181 * MMSE	0.05 (−0.02–0.13)

*Note*: Scaling parameters are reported in Supplemental Table 5.

Abbreviation: CI, confidence interval; MMSE, Mini‐Mental State Examination, MRI, magnetic resonance imaging; SD, standard deviation.

Figure 1 shows the risk of progressing to dementia in the total population (Figure 1A) and the amyloid‐positive subgroup (Figure 1B). All amyloid‐positive (A+) groups had a substantial risk of progression to dementia, with a predicted median time of 7.4 years (95% CI: 6.4–8.1) in A+‐Low, 4.0 years (95% CI: 3.6–4.1) in A+‐Low‐Medium, 2.8 years (95% CI: 2.5–3.0) in A+‐Medium‐High, and 2.0 years (95% CI: 2.0–2.1) in A+‐High.

## 4 DISCUSSION

We refitted the ABIDE model, predicting progression from MCI to dementia, using automated Elecsys CSF measurements. We showed that the model is well calibrated in amyloid‐positive patients and has value for discussing prognosis with patients potentially eligible for treatment. As we found that the risk of progression was high in all amyloid‐positive patients, the model can be used to estimate time to dementia in these patients rather than to predict whether they progress.

The refitted ABIDE model remained largely unchanged, except for a larger interaction effect between pTau181 and Aβ1‐42. This is in line with the higher discriminative value of the pTau181/Aβ1‐42‐ratio measured with the Elecsys assay for determining amyloid‐positivity compared to manual assays using Aβ1‐42.^4^ The ability of the model to discriminate between the level of progression risks of amyloid‐positive participants was limited. This can be attributed to the uniformly high progression risk for amyloid‐positive individuals, while in the total population individuals could be identified who were not likely to progress. New predictors differentiating progression risk in amyloid‐positive patients are needed to improve the predictions in this highly relevant patient population.

### 4.1 Strengths and limitations

The major strength of this study is the combination of multiple cohorts, supporting generalizability to European and USA memory clinic populations. The number of MCI patients also allowed for well‐powered validation of amyloid‐positive individuals. There are a few limitations to this study. First, this study has limited generalizability to non‐Western healthcare settings or the general population due to the limited age range of the included cohorts. Second, the ABIDE model uses CSF and MRI biomarkers and is thus not suitable for use in a primary care setting. Third, as is common when using real‐world data, information on CSF and MRI measurements was not complete and required imputation. Residual bias after imputation cannot be excluded.

## 5 CONCLUSION

We refitted the ABIDE model, an MCI to dementia prediction model, with automated CSF measurements and showed that it is well calibrated for amyloid‐positive patients. The model is well‐suited for use with automated CSF measurements with Elecsys and can be used to support clinical decision making regarding the initiation of ATTs.

## CONFLICT OF INTEREST STATEMENT

P.J. van der Veere, S.J.B. Vos, J. Kornhuber, W. Maier, E. Rüther, Y. Freund‐Levi, A.K. Wallin, I. Kłoszewska, P. Mecocci, B. Vellas, S. Galluzzi, S. Herukka, I. Santana, I. Baldeiras, A. de Mendonca, D. Silva, G. Chetelat, G. Poisnel, P.J. Visser, G. Piñol‐Ripoll, J Berkhof have no disclosures.

Research of C.E.T. is supported by the European Commission (Marie Curie International Training Network, grant agreement No 860197 (MIRIADE) and No 101119596 (TAME), Innovative Medicines Initiatives 3TR (Horizon 2020, grant no 831434) EPND ( IMI 2 Joint Undertaking (JU), grant No. 101034344) and JPND (bPRIDE, CCAD), European Partnership on Metrology, co‐financed from the European Union's Horizon Europe Research and Innovation Programme and by the Participating States (22HLT07 NEuroBioStand), Horizon Europe (PREDICTFTD, 101156175), CANTATE project funded by the Alzheimer Drug Discovery Foundation, Alzheimer Association, Michael J Fox Foundation, Health Holland, the Dutch Research Council (ZonMW), Alzheimer Drug Discovery Foundation, The Selfridges Group Foundation, Alzheimer Netherlands. C.T. is recipient of ABOARD, which is a public‐private partnership receiving funding from ZonMW (#73305095007) and Health∼Holland, Topsector Life Sciences & Health (PPP‐allowance; #LSHM20106). CT is recipient of TAP‐dementia, a ZonMw funded project (#10510032120003) in the context of the Dutch National Dementia Strategy. C.E.T. has research contracts with Acumen, ADx Neurosciences, AC‐Immune, Alamar, Aribio, Axon Neurosciences, Beckman‐Coulter, BioConnect, Bioorchestra, Brainstorm Therapeutics, Celgene, Cognition Therapeutics, EIP Pharma, Eisai, Eli Lilly, Fujirebio, Instant Nano Biosensors, Novo Nordisk, Olink, PeopleBio, Quanterix, Roche, Toyama, Vivoryon. C.E.T. has research contracts with Acumen, ADx Neurosciences, AC‐Immune, Alamar, Aribio, Axon Neurosciences, Beckman‐Coulter, BioConnect, Bioorchestra, Brainstorm Therapeutics, C2N diagnostics, Celgene, Cognition Therapeutics, EIP Pharma, Eisai, Eli Lilly, Fujirebio, Instant Nano Biosensors, Merck, Muna, Novo Nordisk, Olink, PeopleBio, Quanterix, Roche, Toyama, Vaccinex, Vivoryon. She is editor in chief of Alzheimer Research and Therapy, and serves on editorial boards of Molecular Neurodegeneration, Alzheimer's & Dementia, Neurology: Neuroimmunology & Neuroinflammation, Medidact Neurologie/Springer, and is committee member to define guidelines for Cognitive disturbances, and one for acute Neurology in the Netherlands. She has consultancy/speaker contracts for Aribio, Biogen, Beckman‐Coulter, Cognition Therapeutics, Eisai, Eli Lilly, Merck, Novo Nordisk, Novartis, Olink, Roche, Sanofi and Veravas.

W.F. is executive director at Alzheimer Nederland, Amersfoort the Netherlands. In the past, research programs of Wiesje van der Flier have been funded by ZonMW, NWO, EU‐JPND, EU‐IHI, Alzheimer Nederland, Hersenstichting CardioVascular Onderzoek Nederland, Health∼Holland, Topsector Life Sciences & Health, stichting Dioraphte, Noaber foundation, Pieter Houbolt Fonds, Gieskes‐Strijbis fonds, stichting Equilibrio, Edwin Bouw fonds, Pasman stichting, Philips, Biogen MA Inc, Novartis‐NL, Life‐MI, AVID, Roche BV, Eli‐Lilly‐NL, Fujifilm, Eisai, Combinostics. WF is recipient of ABOARD, which is a public‐private partnership receiving funding from ZonMW (#73305095007) and Health∼Holland, Topsector Life Sciences & Health (PPP‐allowance; #LSHM20106). In the past, W.F. has been an invited speaker at Biogen MA Inc, Danone, Eisai, WebMD Neurology (Medscape), NovoNordisk, Springer Healthcare, European Brain Council. WF has been consultant to Oxford Health Policy Forum CIC, Roche, Biogen MA Inc, Eisai, Eli‐Lilly, Owkin France, Nationale Nederlanden Ventures. W.F. has participated in advisory boards of Biogen MA Inc, Roche, and Eli Lilly. W.F. has been member of the steering committee of phase 3 EVOKE/EVOKE+ studies (NovoNordisk). W.F. has been member of the steering committee op phase 3 Trontinemab study (Roche). All funding has been paid to Amsterdam UMC. WF was associate editor of Alzheimer, Research & Therapy in 2020/2021. W.F. was associate editor at Brain 2021‐2025. W.F. is chair of the Scientific Leadership Group of InRAD.

W.F. is member of Supervisory Board (Raad van Toezicht) Trimbos Instituut.

H. Hampel was an employee of Eisai Inc.; however, this article does not represent the opinion of Eisai. HH declares no competing financial interests related to the present article, and his contribution to this article reflects exclusively his academic and scientific expertise. He serves as Reviewing Editor and previously as Senior Associate Editor for the journal Alzheimer's & Dementia, the journal of the Alzheimer's Association. Part of this study was initiated and developed in line with the Alzheimer's Precision Medicine Initiative (APMI) framework.

H.H. is inventor of 11 patents and has received no royalties:

1. In Vitro Multiparameter Determination Method for The Diagnosis and Early Diagnosis of Neurodegenerative Disorders Patent Number: 8916388

2. In Vitro Procedure for Diagnosis and Early Diagnosis of Neurodegenerative Diseases Patent Number: 8298784

3. Neurodegenerative Markers for Psychiatric Conditions Publication Number: 20120196300

4. In Vitro Multiparameter Determination Method for The Diagnosis and Early Diagnosis of Neurodegenerative Disorders Publication Number: 20100062463

5. In Vitro Method for The Diagnosis and Early Diagnosis of Neurodegenerative Disorders Publication Number: 20100035286

6. In Vitro Procedure for Diagnosis and Early Diagnosis of Neurodegenerative Diseases Publication Number: 20090263822

7. In Vitro Method for The Diagnosis of Neurodegenerative Diseases Patent Number: 7547553

8. CSF Diagnostic in Vitro Method for Diagnosis of Dementias and Neuroinflammatory Diseases Publication Number: 20080206797

9. In Vitro Method for The Diagnosis of Neurodegenerative Diseases Publication Number: 20080199966

10. Neurodegenerative Markers for Psychiatric Conditions Publication Number: 20080131921

11. Method for diagnosis of dementias and neuroinflammatory diseases based on an increased level of procalcitonin in CSF: Publication number: United States Patent 10921330.

O. Hansson is an employee of Eli Lilly and Lund University.

S.C. Johnson has received grants from the National Institutes of Health (AG027161 and AG 021155). He has received consulting fees from Lilly, Enigma Biomedical, Alamar and AlzPath.

S. Palmqvist has acquired research support (for the institution) from Avid and ki elements through ADDF. In the past 2 years, he has received consultancy/speaker fees from Bioartic, Biogen, Eisai, Eli Lilly, Novo Nordisk, and Roche.

I.S. van Maurik received funding form ZonMW and STI‐MAG and a consulting fee from Roche (paid to the institution).

M. Tsolaki received funding from Altoida, Eisai and Ionian University.

S. Lovestone is currently employed at J&J.

A.C. van Harten has received funding from ZonMW and Alzheimer Nederland (WE.06‐2021‐06) and has received consulting fees from Lilly.

Giovanni B. Frisoni has received funding through the Private Foundation of Geneva University Hospitals from: A.P.R.A. – Association Suisse pour la Recherche sur la Maladie d'Alzheimer, Genève; Fondation Segré, Genève; Ivan Pictet, Genève; Race Against Dementia Foundation, London, UK; Fondation Child Care, Genève; Fondation Edmond J. Safra, Genève; Fondation Minkoff, Genève; Fondazione Agusta, Lugano; McCall Macbain Foundation, Canada; Nicole et René Keller, Genève; Fondation AETAS, Genève. He has received funding through the University of Geneva or Geneva University Hospitals: for IISSs from ROCHE Pharmaceuticals OM Pharma EISAI Pharmaceuticals Biogen Pharmaceuticals and Novo Nordisk; for competitive research projects from: H2020, Innovative Medicines Initiative (IMI), IMI2, Swiss National Science Foundation, and VELUX Foundation. GBF has received payment or honoraria for lectures, presentations, speakers bureaus, manuscript writing, or educational events from: Biogen, Roche, Novo Nordisk, Biogen, GE HealthCare. All through my institution.

E. Stormrud has performed contract research for C2N diagnostics, Fujirebio, GE healthcare and Roche Diagnostics. With research support paid to his institution.

F. Barkhof has research agreements with ADDI, Merck, Biogen, GE Healthcare, Roche. He has received consulting fees from Roche, Celltrion, Rewind Therapeutics, Merck, Bracco. He is on the steering committee or Data Safety Monitoring Board member for Biogen, Merck, Eisai and Prothena. He is on the advisory board member for Combinostics, Scottish Brain Sciences, Alzheimer Europe. He is co‐founder and shareholder of Queen Square Analytics LTDb.

L. Spiru has received speaking fees from sunwavePharma, Sanoffi, PharmaNord and Unicredit Banl. She has received payments for expert testimony from sunwavePharma, Sanoffi and PharmaNord. She participated on a data safety monitoring board or advisory board for Sanoffi and GCLS. She fulfilled a leadership or fiduciary role in other board, society, committee or advocacy group, paid or unpaid at GCLS, HES‐GE and EWA.

J. Wiltfang has received a grant from BMBF (13GW0379B). He has received consulting fees from Immungenetics, Noselab and Roboscreening. He has received speaking fees from Beeijing Yibai Science and Technology Ltd., Gloryren, Janssen Cilag, Pfizer, Med Update GmbH, Roche Pharma, Lilly. He has filled the following patents PCT/EP 2011 001724, PCT/EP 2015 052945. He has participated on a Data Safety Monitoring Board or Advisory Board for Biogen, Abbott, Boehringer Ingelheim, Lilly, MSD Sharp & Dohme, and Roche. He has fulfilled a leadership or fiduciary role in other board, society, committee or advocacy group, paid or unpaid at AGNP, DGLN, DGPPN, Deutsche Hirnliga, CSF‐society.

O. Peters has received consulting/speaking fees from Biogen, Lilly, Eisai, Grifols, Noselab, NovoNordisk, Prinnovation, and Roche. He is a board member of the German Dementia Competence Network and Hirnliga.

L. Froelich has received grants from Hoffmann‐LaRoche and Hector II Foundation, Dietmar Hopp Foundation, EU‐H2020‐2017 (Grant no. 779237); EU‐IMI‐2019 (grant no. 806999); EU‐H2022 (Grant No. 101120706), EU‐H2024 (Grant no. 101156500). He has received royalties or licenses for Avanex, Biogen, BioVie, Bristol‐Myers Squibb, Charles‐River Ass., Eli Lilly, Eisai, GE Healthcare, Grifols, Janssen‐Cilag, Janssen Research, Neurimmune, Noselab, NovoNordisk, Roche, TauRX, Schwabe. He has received consulting fees from Eli Lilly, Eisai, DerCampus, Medscape, Medfora, FOMF, NovoNordisk, Roche, Schwabe. He has participated on a Data Safety Monitoring Board or Advisory Board at Neuroscios, ReMynd, Otsuka/Avanir, Vivoryon. He has performed a leadership or fiduciary role in other board, society, committee or advocacy group, paid or unpaid at German Society of Psychiatry, Psychotherapy and Nervous Diseases (Guideline committee), European Alzheimer Disease Consortium, German Network of Memory Clinics, Alzheimer Europe, Alzheimer Society Baden‐Württemberg. Author disclosures are available in the Supporting Information.

## STANDARD PROTOCOL APPROVALS, REGISTRATIONS, AND PATIENT CONSENTS

The study protocol of the ADC, under which this study was conducted, was approved by the ethical review board of the VU University Medical Center (2016.061).

## Supporting information



Supporting Information

Supporting Information

Supporting Information

## Data Availability

Data of the included cohorts may be shared upon request. Code used to perform the analyses may be made available upon request.
