# Supplementary material for: Revising the ABIDE MCI to dementia prediction model for automated cerebrospinal fluid assays
Source: Alzheimers Dement. 2026 Feb 9;22(2):e71192. doi: 10.1002/alz.71192 (PMC12884196; doi:10.1002/alz.71192)
Supplement: Supplementary file 1 — Supporting Information [file ALZ-22-e71192-s002.docx]

**Supplemental Materials** **Revising the ABIDE MCI to dementia prediction model for automated cerebrospinal fluid assays**

**Supplemental Methods**

CSF Aβ1-42 measurements in ADC before 2018 were performed using Innotest and were drift correct.^1^ CSF Aβ1-42 and pTau181 measurements were converted between platforms to represent the value the measurements likely would have taken if they were performed using Elecsys. The cross formula for conversion from Innotest to Elecsys was published before by Willems et al. (2018),^2^ and the formula for conversion from Luminex to Elecsys was included in the supplemental materials of Maurik et al. (2019).^3^

Participants were said to be amyloid-positive based on the unconverted CSFS values. For Elecsys, a pTau181/Aβ1-42-ratio cut-off of more than or equal to 0.020 indicated amyloid-positivity.^4^ For Luminex a Aβ1-42 value below 250 pg/ml indicated amyloid-positivity.^5^ For Innotest, a (drift-corrected) value below 813 indicated amyloid-positivity.^1^

Missing CSF and CSF and MRI variables were imputed with multiple imputation using chained equations based on predictive mean matching in twenty imputed datasets. Variables used in the identification of donors were selected based on a minimal correlation of 0.05 with the variable being imputed. The distribution of imputed values and convergence were assessed visually. Parameter estimates were pooled across the imputation sets. In the cross-validations, predicted risk of participants in the test cohort was averaged over the imputed datasets. Similarly, the baseline hazard estimated by the models on the training cohorts was averaged over the imputed datasets and used for the predictions in the test cohort.

Mean and standard deviations used to scale all predictors are included in Supplemental Table 5.

**Supplemental Results**

**Stratified by cohort, predicted and observed progression probabilities was slightly underestimate in ADC and overestimated in ADNI after five years (Supplemental Figure 3). Stratified by CSF platform, predicted and observed MCI to dementia progression probability were similar for the Elecsys ad Luminex assays with a slight underestimation of risk for the Innotest assay (Supplemental Figure 4). However, only 41 participants with Luminex CSF measurements were included, leading to substantial uncertainty in the observed MCI to dementia progression in the group with Luminex measurements.**

**Supplemental Table 1**: Cohort characteristics

| Characteristic | ADC | ADNI | BioFINDER-1 | Lleida | NACC | W-ADRC |
| --- | --- | --- | --- | --- | --- | --- |
| N | 689 | 544 | 212 | 88 | 63 | 8 |
| Baseline data collection period | 1999-2023 | 2005-2022 | 2010-2014 | 2018-2019 | 2005-2015 |  |
| Study design | Observational cohort | Observational cohort | Observational cohort | Observational cohort | Observational cohort | Observational cohort |
| Setting | Tertiary memory clinic | Research cohort | Memory clinic | Memory clinic | Research cohort | Research cohort |
| Cohort specific inclusion criteria | Referred with memory clinic, MCI diagnoses conformed to the NIA-AA criteria^6^ | Between 55 and 90 years old, memory complaints, MMSE between 24 and 30, clinical dementia rating of 0.5 with an abnormal memory box score, did not qualify for diagnosis of dementia^7^ | Referred to memory clinic due to memory complaints, between 60 and 80 years old, MMSE 24-30, do not fulfil criteria of dementia^8^ | Presented at memory clinic, neurologist requested CSF analysis, MCI diagnosis based on NIA-AA^6^ | Varying by Alzheimer Disease Centre, stable health status, met core clinical criteria of MCI^6^ | Age over 45yr, fluent in English^9^ |
| Follow-up structure | Yearly clinical follow-up | Six to twelve month intervals | Yearly follow-up | Approximately six monthly follow-up | Yearly follow-up | Yearly follow-up |
| Criterium for progression to dementia | Criteria used differed based on likely etiology^6, 10-13^ | NIA-AA criteria for “probable AD”^6^ | DSM-5 for major neurocognitive disorder^14^ | NIA-AA criteria for “probable AD”^6^ | Criteria fulfil the NIA-AA criteria for all-cause dementia^6^ | Criteria fulfil the NIA-AA criteria for all-cause dementia^6^ |
| MRI quantification method | Freesurfer 7.1 | FreeSurfer version 4.3 for ADNI 1 1.5T MRI data, version 5.1 for the ADNI GO/2 and ADNI 1 3T MRI data, version 6.0 for ADNI 3 3T MRI data.^15^ | Freesurfer 7.2.0^16^ | - | Manual segmentation | - |
| MRI standardisation performed | Yes, using the ComBat procedure | Yes, following the EADC-ADNI harmonized protocol^17^ | No, but all scans were performed using a 3T MRI Philips Achieva scanner^16^ | - | Yes, following the EADC-ADNI harmonized protocol^17^ | - |
| MRI data availability | 82% | 92% | 93% | 0% | 49% | 0% |
| CSF quantification method | Innotest until 2018, Elecsys afterwards | Elecsys | Elecsys | Elecsys | Luminex | Elecsys |
| CSF data availability | 82% | 66% | 92% | 100% | 65% | 100% |
| Other | - | - | - | - | Data from three centres was included | The MOCA was performed at baseline, scores were converted to an MMSE score^18^ |

Cohort characteristics of the included EMIF cohorts are outlined in the supplemental material of Maurik et al. (2019).^3^

Abbreviations: SD = standard deviation, MMSE = mini-mental state examination, MOCA= Montreal Cognitive Assessment, MRI= magnetic resonance imaging, ADC= Amsterdam Dementia Cohort, ADNI = Alzheimer’s Disease Neuroimaging Initiative, BioFINDER = Biomarkers For Identifying Neurodegenerative Disorders Early and Reliably Study, EMIF-AD = European Medical Information Framework for [Alzheimer's Disease](https://www.sciencedirect.com/topics/pharmacology-toxicology-and-pharmaceutical-science/alzheimers-disease), NACC = National Alzheimer’s Coordinating Centre, W-ADRC = Wisconsin Alzheimer’s Disease Research Centre

**Supplemental Table 2**: Baseline characteristics stratified on inclusion in earlier model, newly included in the model, or included with additional follow-up data

|  |  | New data included | |
| --- | --- | --- | --- |
|  | Previously included  (n = 1741) | New participants  included  (n = 478) | Additional follow-up  (n = 194) |
| Follow-up time (years) - median (IQR) | 2.0 (1.2 to 3.0) | 2.1 (1.1 to 3.6) | 6.0 (3.4 to 8.9) |
| Participants progression to dementia, n (%) | 791 (45.4) | 196 (41.0) | 47 (24.2) |
| Age at baseline (years) - mean ± SD | 69 ± 8 | 70 ± 8 | 70 ± 9 |
| Female - n (%) | 957 (55.0) | 292 (61.1) | 124 (63.9) |
| MMSE at baseline - mean ± SD | 26.8 ± 2.2 | 26.6 ± 2.4 | 27.6 ± 1.8 |
| Amyloid Beta 1-42 (pg/ml) - median (IQR) | 864 (608 to 1302) | 745 (553 to 1142) | 976 (740 to 1465) |
| Phosporylated Tau (pg/ml) - median (IQR) | 23.8 (15.8 to 34.2) | 24.2 (16.7 to 34.5) | 19.9 (15.5 to 30.4) |
| Amyloid positive - n (%) | 687 (39.5) | 240 (50.2) | 66 (34.0) |
| MRI Hippocampal volume (ml) - mean ± SD | 6.7 ± 1.2 | 7.0 ± 1.2 | 7.2 ± 1.1 |
| CSF platform - n (%) |  |  |  |
| Elecsys | 431 (39.9) | 271 (77.4) | 73 (50.0) |
| Innotest | 648 (60.1) | 38 (10.9) | 73 (50.0) |
| Luminex | - | 41 (11.7) | - |
| Cohorts - n (%) |  |  |  |
| EMIF | 809 (46.5) | - | - |
| ADC | 356 (20.4) | 243 (50.8) | 90 (46.4) |
| ADNI | 364 (20.9) | 76 (15.9) | 104 (53.6) |
| BioFINDER-1 | 212 (12.2) | - | - |
| Lleida | - | 88 (18.4) | - |
| USA Other† | - | 71 (14.9) | - |

†Contains the NACC and W-ADRC cohorts. “-“ was used if no data was present.

Abbreviations: SD = standard deviation, MMSE = Mini-Mental State Examination, MRI= magnetic resonance imaging, ADC= Amsterdam Dementia Cohort, ADNI = Alzheimer’s Disease Neuroimaging Initiative, BioFINDER = Biomarkers For Identifying Neurodegenerative Disorders Early and Reliably Study, EMIF-AD = European Medical Information Framework for [Alzheimer's Disease](https://www.sciencedirect.com/topics/pharmacology-toxicology-and-pharmaceutical-science/alzheimers-disease), NACC = National Alzheimer’s Coordinating Centre, W-ADRC = Wisconsin Alzheimer’s Disease Research Centre

**Supplemental Table 3**: Baseline characteristics of amyloid-positive participants

|  | | Cohorts | | | | | |
| --- | --- | --- | --- | --- | --- | --- | --- |
|  | Total (n = 998) | ADC (n = 344) | ADNI  (n = 225) | EMIF  (n = 224) | BioFINDER-1  (n = 109) | LLeida  (n = 55) | USA Other (n = 41)† |
| Follow-up time (years) - median (IQR) | 2.1 (1.3 to 3.1) | 2.2 (1.3 to 3.4) | 2.0 (1.1 to 3.2) | 2.0 (1.3 to 3.0) | 2.3 (2.0 to 4.1) | 1.0 (0.7 to 3.1) | 1.9 (1.1 to 3.2) |
| Participants progressed to dementia, n (%) | 585 (58.6) | 195 (56.7) | 150 (66.7) | 96 (42.9) | 79 (72.5) | 40 (72.7) | 25 (61.0) |
| Age at baseline (years) - mean ± SD | 70 ± 8 | 66 ± 7 | 74 ± 7 | 68 ± 8 | 72 ± 5 | 74 ± 6 | 74 ± 9 |
| Female - n (%) | 557 (55.8) | 193 (56.1) | 133 (59.1) | 126 (56.2) | 60 (55.0) | 24 (43.6) | 21 (51.2) |
| MMSE at baseline - mean ± SD | 26.6 ± 2.3 | 26.5 ± 2.3 | 27.1 ± 1.8 | 26.5 ± 2.4 | 26.8 ± 1.7 | 24.8 ± 3.0 | 26.9 ± 2.1 |
| Amyloid Beta 1-42 (pg/ml) - median (IQR) | 666 (512 to 839) | 696 (555 to 861) | 638 (526 to 776) | 610 (418 to 846) | 676 (500 to 830) | 596 (477 to 717) | 659 (471 to 1105) |
| Phosporylated Tau (pg/ml) - median (IQR) | 29.7 (21.6 to 39.7) | 30.4 (24.3 to 40.1) | 32.4 (25.0 to 41.8) | 24.4 (13.4 to 36.5) | 29.4 (22.0 to 36.3) | 27.4 (19.7 to 37.4) | 30.4 (18.1 to 40.0) |
| MRI Hippocampal volume (ml) - mean ± SD | 6.6 ± 1.1 | 7.0 ± 1.0 | 6.3 ± 1.0 | 6.6 ± 1.1 | 6.0 ± 0.9 | - | 6.4 ± 0.8 |
| CSF platform - n (%) | | | | | | | |
| Elecsys | 622 (62.3) | 226 (65.7) | 225 (100) | - | 109 (100) | 55 (100) | 7 (17.1) |
| Innotest | 342 (34.3) | 118 (34.3) | - | 224 (100) | - | - | - |
| Luminex | 34 (3.4) | - | - | - | - | - | 34 (82.9) |

†Contains the NACC and W-ADRC cohorts. “-“ was used if no data was present.

Abbreviations: SD = standard deviation, MMSE = Mini-Mental State Examination, MRI= magnetic resonance imaging, ADC= Amsterdam Dementia Cohort, ADNI = Alzheimer’s Disease Neuroimaging Initiative, BioFINDER = Biomarkers For Identifying Neurodegenerative Disorders Early and Reliably Study, EMIF-AD = European Medical Information Framework for [Alzheimer's Disease](https://www.sciencedirect.com/topics/pharmacology-toxicology-and-pharmaceutical-science/alzheimers-disease), NACC = National Alzheimer’s Coordinating Centre, W-ADRC = Wisconsin Alzheimer’s Disease Research Centre

**Supplemental Table 4**: Supplement table comparing new model parameters with centring used in Van Maurik 2019

|  | ABIDE 2019 regression coefficients (95% CI) |  | Refitted regression coefficients using old centring (95% CI) |  | Refitted partial regression coefficients with scaling (95% CI) |
| --- | --- | --- | --- | --- | --- |
| Amyloid β, log ng/ml | −0·53 (−0·63 to −0·41) |  | -0.60  (-0.74 to -0.46) |  | -0.33 (-0.40 to -0.25) |
| Phosphorylated tau, log ng/ml | 0·62 (0·44 to 0·80) |  | 0.58  (0.43 to 0.73) |  | 0.33 (0.25 to 0.41) |
| Hippocampal volume, cm^3^ | −0·42 (−0·52 to −0·32) |  | -0.37  (-0.45 to -0.29) |  | -0.37 (-0.45 to -0.29) |
| Age, year | −0·007 (−0·02 to 0·007) |  | -0.01  (-0.02 to 0.002) |  | -0.05 (-0.12 to 0.03) |
| MMSE, point increase | −0·11 (−0·15 to −0·07) |  | -0.10  (-0.13 to -0.08) |  | -0.23 (-0.30 to -0.17) |
| Interactions |  |  |  |  |  |
| Amyloid β * phosphorylated tau | 0·18 (−0·02 to 0·38) |  | 0.25 (-0.09 to 0.58) |  | 0.07 (-0.03 to 0.17) |
| Amyloid β * age | 0·017 (−0·002 to 0·04) |  | 0.01  (-0.01 to 0.03) |  | 0.04 (-0.03 to 0.12) |
| Phosphorylated * MMSE | 0·093 (0·02 to 0·18) |  | 0.04  (-0.02 to 0.10) |  | 0.05 (-0.02 to 0.13) |

**Supplemental Table 5**: Exact mean and standard deviation used for scaling variables

|  | Mean |  | Standard deviation |
| --- | --- | --- | --- |
| Amyloid β, log ng/ml | 6.767684 |  | 0.5430144 |
| Phosphorylated tau, log ng/ml | 3.129617 |  | 0.5568504 |
| Hippocampal volume, cm^3^ | 6.796858 |  | 1.167452 |
| Age, year | 69.4973 |  | 8.221856 |
| MMSE, point increase | 26.8408 |  | 2.256473 |


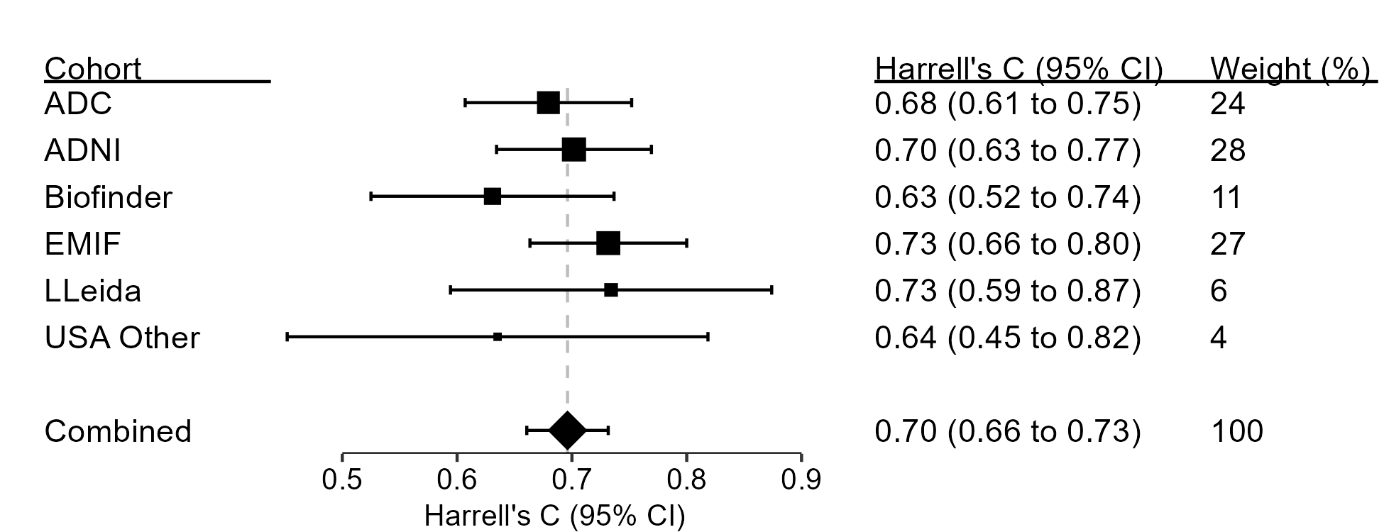


**Supplemental Figure 1**: Leave-one-cohort-out cross-validation results by cohort

Points were sized to the inverse of the variance. Cohorts were ordered alphabetically. Harrell’s C’s were pooled using inverse variance weighting. Abbreviations: CI = confidence interval, ADC = Amsterdam Dementia Cohort, ADNI = Alzheimer’s Disease Neuroimaging Initiative, BioFINDER = Biomarkers For Identifying Neurodegenerative Disorders Early and Reliably Study, EMIF = European Medical Information Framework for [Alzheimer's Disease](https://www.sciencedirect.com/topics/pharmacology-toxicology-and-pharmaceutical-science/alzheimers-disease)


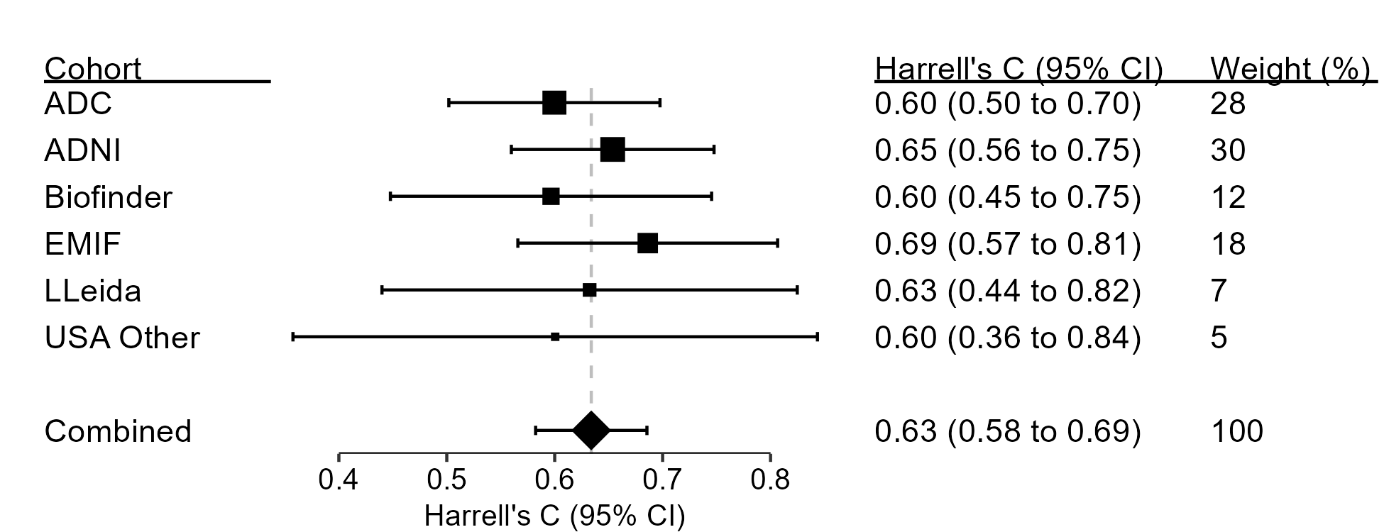


**Supplemental Figure 2**: Leave-one-cohort-out cross-validation results by cohort for amyloid-positive participants

Points were sized to the inverse of the variance. Cohorts were ordered alphabetically. Harrell’s C’s were pooled using inverse variance weighting. Abbreviations: CI = confidence interval, ADC = Amsterdam Dementia Cohort, ADNI = Alzheimer’s Disease Neuroimaging Initiative, BioFINDER = Biomarkers For Identifying Neurodegenerative Disorders Early and Reliably Study, EMIF = European Medical Information Framework for [Alzheimer's Disease](https://www.sciencedirect.com/topics/pharmacology-toxicology-and-pharmaceutical-science/alzheimers-disease)


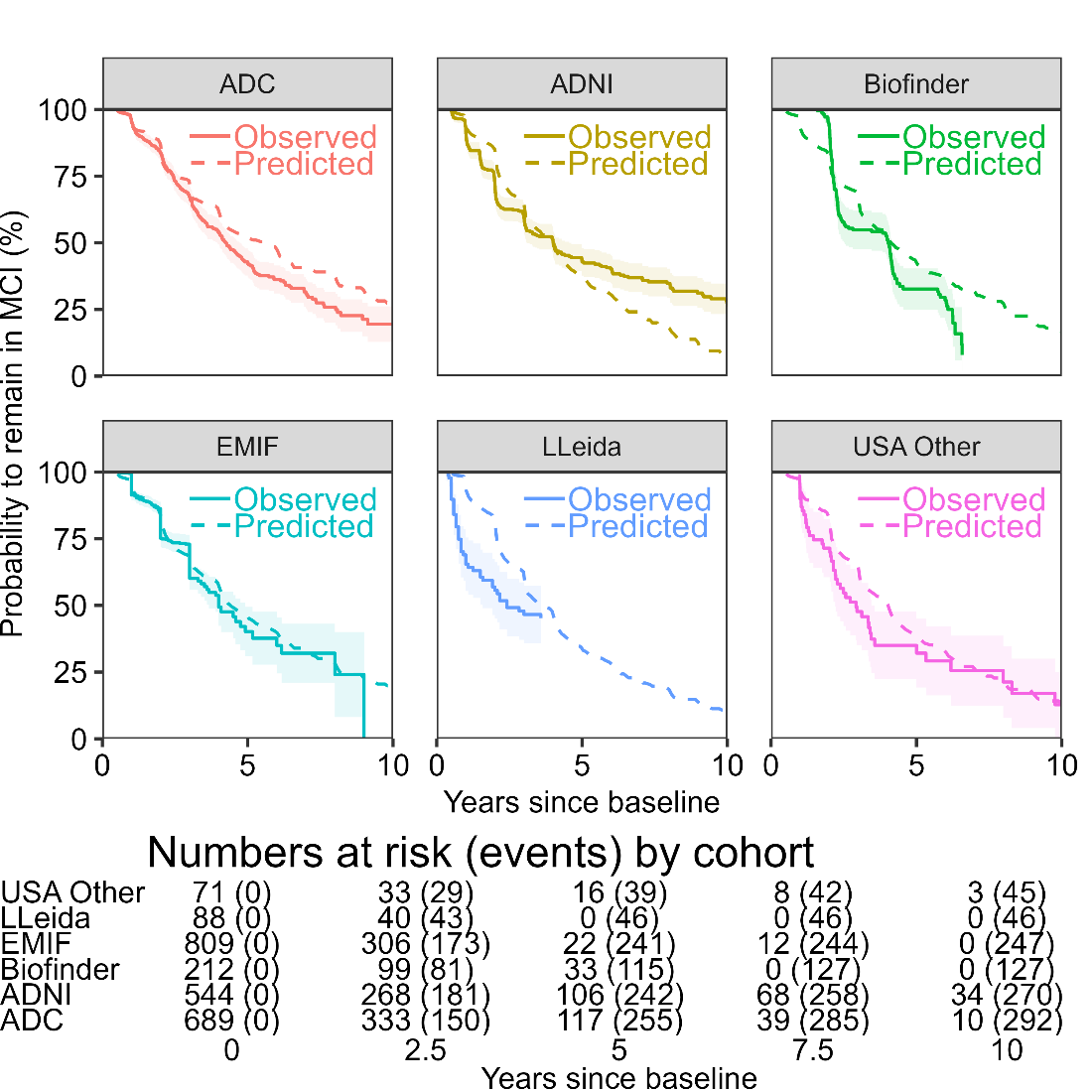


**Supplemental Figure 3**: Predicted risk groups by the ABIDE model stratified by cohort

The predicted survival curves are the average predicted survival probabilities of individuals in each risk group. Leave-on-cohort-out cross-validation was used to make the predictions where iteratively one cohort was selected as “test” set in which predictions were made using a model develop in the other cohorts. The shaded areas correspond to the 95% confidence intervals of the observed survival probabilities. Abbreviations: ADC = Amsterdam Dementia Cohort, ADNI = Alzheimer’s Disease Neuroimaging Initiative, BioFINDER = Biomarkers For Identifying Neurodegenerative Disorders Early and Reliably Study, EMIF = European Medical Information Framework for [Alzheimer's Disease](https://www.sciencedirect.com/topics/pharmacology-toxicology-and-pharmaceutical-science/alzheimers-disease).

**Supplemental Figure 4**: Predicted risk groups by the ABIDE model stratified by CSF assay


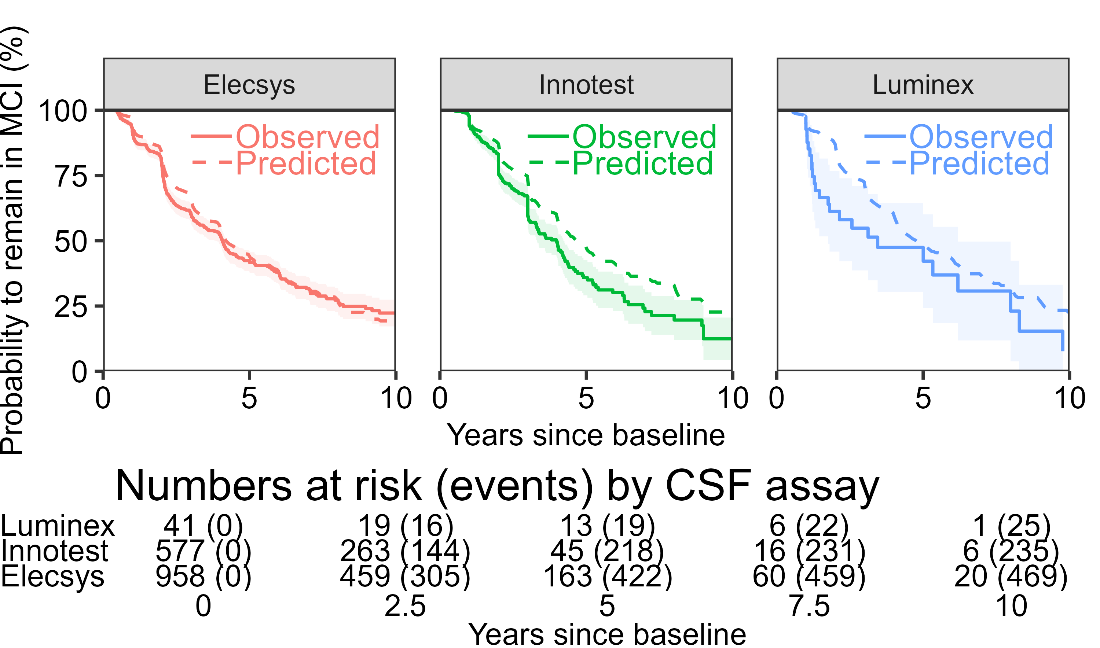


The predicted survival curves are the average predicted survival probabilities of individuals in each risk group. Leave-one-assay-out cross-validation was used to make the predictions where iteratively one assay was selected as “test” set in which predictions were made using a model develop in the other assays. The shaded areas correspond to the 95% confidence intervals of the observed survival probabilities.

**References**

1. Tijms BM, Willemse EAJ, Zwan MD, Mulder SD, Visser PJ, van Berckel BNM, et al. Unbiased Approach to Counteract Upward Drift in Cerebrospinal Fluid Amyloid-beta 1-42 Analysis Results. Clin Chem. 2018;64(3):576-85.

2. Willemse EAJ, van Maurik IS, Tijms BM, Bouwman FH, Franke A, Hubeek I, et al. Diagnostic performance of Elecsys immunoassays for cerebrospinal fluid Alzheimer's disease biomarkers in a nonacademic, multicenter memory clinic cohort: The ABIDE project. Alzheimer's & Dementia: Diagnosis, Assessment & Disease Monitoring. 2018;10(1):563-72.

3. van Maurik IS, Vos SJ, Bos I, Bouwman FH, Teunissen CE, Scheltens P, et al. Biomarker-based prognosis for people with mild cognitive impairment (ABIDE): a modelling study. Lancet Neurol. 2019;18(11):1034-44.

4. van Harten AC, Wiste HJ, Weigand SD, Mielke MM, Kremers WK, Eichenlaub U, et al. Detection of Alzheimer's disease amyloid beta 1-42, p-tau, and t-tau assays. Alzheimers Dement. 2022;18(4):635-44.

5. Shaw LM, Waligorska T, Fields L, Korecka M, Figurski M, Trojanowski JQ, et al. Derivation of cutoffs for the Elecsys(®) amyloid β (1-42) assay in Alzheimer's disease. Alzheimers Dement (Amst). 2018;10:698-705.

6. McKhann GM, Knopman DS, Chertkow H, Hyman BT, Jack CR, Jr., Kawas CH, et al. The diagnosis of dementia due to Alzheimer's disease: recommendations from the National Institute on Aging-Alzheimer's Association workgroups on diagnostic guidelines for Alzheimer's disease. Alzheimers Dement. 2011;7(3):263-9.

7. Petersen RC, Aisen PS, Beckett LA, Donohue MC, Gamst AC, Harvey DJ, et al. Alzheimer's Disease Neuroimaging Initiative (ADNI): clinical characterization. Neurology. 2010;74(3):201-9.

8. study SB. BioFINDER: Population & Study Design [Available from: <https://biofinder.se/one/population-study-design/>.

9. Ma Y, Mora Pinzon MC, Buckingham WR, Bersch AJ, Powell WR, LeCaire TJ, et al. Comparison of sample characteristics of Wisconsin Alzheimer's Disease Research Center participants with the Wisconsin state population-An evaluation of the recruitment effort. Alzheimers Dement (N Y). 2025;11(1):e70036.

10. McKeith IG, Dickson DW, Lowe J, Emre M, O'Brien JT, Feldman H, et al. Diagnosis and management of dementia with Lewy bodies: third report of the DLB Consortium. Neurology. 2005;65(12):1863-72.

11. Neary D, Snowden JS, Gustafson L, Passant U, Stuss D, Black S, et al. Frontotemporal lobar degeneration: a consensus on clinical diagnostic criteria. Neurology. 1998;51(6):1546-54.

12. Román GC, Tatemichi TK, Erkinjuntti T, Cummings JL, Masdeu JC, Garcia JH, et al. Vascular dementia: diagnostic criteria for research studies. Report of the NINDS-AIREN International Workshop. Neurology. 1993;43(2):250-60.

13. Albert MS, DeKosky ST, Dickson D, Dubois B, Feldman HH, Fox NC, et al. The diagnosis of mild cognitive impairment due to Alzheimer's disease: recommendations from the National Institute on Aging-Alzheimer's Association workgroups on diagnostic guidelines for Alzheimer's disease. Alzheimers Dement. 2011;7(3):270-9.

14. American Psychiatric Association. Diagnostic and Statistical Manual of Mental Disorders. 5th ed. Washington, DC2013.

15. Jack CR, Jr., Arani A, Borowski BJ, Cash DM, Crawford K, Das SR, et al. Overview of ADNI MRI. Alzheimers Dement. 2024;20(10):7350-60.

16. Seidu NM, Kern S, Sacuiu S, Sterner TR, Blennow K, Zetterberg H, et al. Association of CSF biomarkers with MRI brain changes in Alzheimer's disease. Alzheimer's & Dementia: Diagnosis, Assessment & Disease Monitoring. 2024;16(1):e12556.

17. Frisoni GB, Jack CR, Jr., Bocchetta M, Bauer C, Frederiksen KS, Liu Y, et al. The EADC-ADNI Harmonized Protocol for manual hippocampal segmentation on magnetic resonance: evidence of validity. Alzheimers Dement. 2015;11(2):111-25.

18. Saczynski JS, Inouye SK, Guess J, Jones RN, Fong TG, Nemeth E, et al. The Montreal Cognitive Assessment: Creating a Crosswalk with the Mini-Mental State Examination. J Am Geriatr Soc. 2015;63(11):2370-4.
